# Metabotropic glutamate receptors as a new therapeutic target for malignant gliomas

**DOI:** 10.18632/oncotarget.15299

**Published:** 2017-02-12

**Authors:** Mery Stefani Leivas Pereira, Fábio Klamt, Chairini Cássia Thomé, Paulo Valdeci Worm, Diogo Losch de Oliveira

**Affiliations:** ^1^ Department of Biochemistry, Laboratory of Cellular Neurochemistry, Instituto de Ciências Básicas da Saúde, Universidade Federal do Rio Grande do Sul, Porto Alegre RS, Brazil; ^2^ Department of Biochemistry, Laboratory of Cellular Biochemistry, Instituto de Ciências Básicas da Saúde, Universidade Federal do Rio Grande do Sul, Porto Alegre RS, Brazil; ^3^ Department of Neurosurgery, Cristo Redentor Hospital – GHC – Porto Alegre RS, Brazil; ^4^ Department of Neurosurgery, São José Hospital, Complexo Hospitalar Santa Casa, Porto Alegre RS, Brazil

**Keywords:** mGluR, glioblastoma, brain cancer

## Abstract

Metabotropic glutamate receptors (mGluR) are predominantly involved in maintenance of cellular homeostasis of central nervous system. However, evidences have suggested other roles of mGluR in human tumors. Aberrant mGluR signaling has been shown to participate in transformation and maintenance of various cancer types, including malignant brain tumors. This review intends to summarize recent findings regarding the involvement of mGluR-mediated intracellular signaling pathways in progression, aggressiveness, and recurrence of malignant gliomas, mainly glioblastomas (GBM), highlighting the potential therapeutic applications of mGluR ligands. In addition to the growing number of studies reporting mGluR gene or protein expression in glioma samples (resections, lineages, and primary cultures), pharmacological blockade *in vitro* of mGluR1 and mGluR3 by selective ligands has been shown to be anti-proliferative and anti-migratory, decreasing activation of MAPK and PI3K pathways. In addition, mGluR3 antagonists promoted astroglial differentiation of GBM cells and also enabled cytotoxic action of temozolomide (TMZ). mGluR3-dependent TMZ toxicity was supported by increasing levels of MGMT transcripts through an intracellular signaling pathway that sequentially involves PI3K and NF-?B. Further, continuous pharmacological blockade of mGluR1 and mGluR3 have been shown to reduced growth of GBM tumor in two independent *in vivo* xenograft models. In parallel, low levels of mGluR3 mRNA in GBM resections may be a predictor for long survival rate of patients. Since several Phase I, II and III clinical trials are being performed using group I and II mGluR modulators, there is a strong scientifically-based rationale for testing mGluR antagonists as an adjuvant therapy for malignant brain tumors.

## INTRODUCTION

Gliomas are the most common type of primary brain tumor and are often fast growing with a poor prognosis for patient [1]. The World Health Organization (WHO) classified gliomas in 2016 using molecular parameters in addition to their histological and immunohistochemical resemblance to presumed cells of origin and graded them by increasing degrees of undifferentiation, anaplasia, and aggressiveness (*i.e*., mitotic figures, necrosis, and vascular endothelial hyperplasia) [2, 3]. High-grade gliomas represent 60-75% of all cases and include grade III anaplastic astrocytoma, anaplastic oligodendroglioma, mixed anaplastic oligoastrocytoma, and grade IV glioblastoma [4]. Glioblastoma (GBM) is the most common malignant primary brain tumor and is one of the most lethal human cancers [2, 5]. Recently, GBM was classified in three groups: (1) GBM, IDH-wildtype (about 90% of cases, generally corresponds to the clinical definition of primary GBM and is prevalent in patients over 55 years); (2) GBM, IDH-mutant (about 10 % of cases, corresponds closely to so-called secondary GBM and preferentially arises in younger patients); and (3) GBM, NOS (a diagnosis that is reserved for those tumors for which full IDH evaluation cannot be performed) [3]. In United States, GBM accounts for 15.1% of all primary brain tumors, 46.1% of primary malignant brain tumors, and its annual incidence is 3.2 per 100,000 people (or 10,787 new cases diagnosed per year) [6]. Even though GBM may develop at any age, it is more common in elderly with a higher incidence rate in ages between 75 to 84 years (15.24 new cases per 100,000 people between these ages per year). In addition, GBM is 1.6 times more common in males and its incidence rate is 2 times higher among caucasians [6].

Regarding histology, GBM is characterized by considerable cellularity and mitotic activity, vascular proliferation, and necrosis [7]. Because GBM cells vary in size and shape, *i.e*., they are pleomorphic, this glioma was frequently called *glioblastoma multiforme*, a term no longer in use. From a molecular point of view, GBM is a highly heterogeneous tumor [8]. Genome-wide expression studies have revealed 4 transcriptional subclasses of GBM, displaying features reminiscent of distinct cell types: classical, mesenchymal, proneural, and neural [9, 10]. Classical subclass typically displays chromosome 7 amplifications, chromosome 10 deletions, *EGFR* amplification, *EGFR* mutations, and *Ink4a/ARF* locus deletion. Mesenchymal subclass displays a high frequency of *NF1* mutation/deletion, high expression of *CHI3L1*, *MET*, and genes involved in tumor necrosis factor and nuclear factor-κB (NF-κB) pathways. Proneural GBM is characterized by alterations of *PDGFRA* and mutations in *IDH1* and *TP53*, sharing gene expression features with low-grade gliomas and secondary GBM (*i.e*., low-grade gliomas later recurred as GBM). Neural subclass is characterized by expression of neuronal markers. Many molecular abnormalities and mutations overlap across transcriptional subclasses, for example *PTEN* loss, and a large number of very rare mutations have been described [11, 12].

Although GBM is typically confined to Central Nervous System (CNS) and rarely performing metastases in distant organs, this and other malignant gliomas are highly invasive, infiltrating surrounding brain parenchyma [5]. After initial diagnosis, standard treatment for GBM consists of maximal surgical resection [13, 14]. This practice aims to relieve mass effect, achieve cytoreduction, and provide adequate tissue for histologic and molecular tumor characterization. Although surgical resection can greatly reduce tumor bulk, complete tumor excision is frequently not reached due to infiltrative nature of GBM cells [15]. After surgical resection, adjuvant radiotherapy combined with chemotherapy should be considered for all patients. A radiotherapy dose of 60 Gy is frequently used [13]. In addition, the DNA alkylating agent named temozolomide (TMZ) is orally administered as first-line chemotherapy [5, 16]. This regimen is supported by a randomized phase III study [17], which demonstrated TMZ increased median survival to 15 months *versus* 12 months with radiotherapy alone (hazard ratio - HR = 0.63; *P* < .001). Two-year survival rate was also increased: 27% for chemotherapy plus radiotherapy *versus* 10% for radiotherapy alone [17]. Alternatively, biodegradable polymers containing the alkylating agent carmustine (BCNU) can be implanted into tumor bed after surgical resection. Nevertheless, a phase III trial has indicated a modest survival benefit of this regimen [18]. A humanized vascular endothelial growth factor (VEGF) monoclonal antibody named bevacizumab had been recently introduced as first-line monotherapy for progressive GBM [19]. Approval of bevacizumab by U.S. Food and Drug Administration was based on improvement of radiologic response rates observed in two single-arm or noncomparative phase II trials [20, 21]. However, two recent multicenter, phase III, randomized, double-blind, placebo-controlled trials [22, 23], have demonstrated bevacizumab increased median progression-free survival (10.6 *vs*. 6.2 months, HR: 0.64, *p* < 0.0001 [22]; 10.7 *vs*. 7.3 months, HR: 0.79, *p* = 0.004 [23]) but not overall survival of patients (16-17 months).

Although radiotherapy and chemotherapy improve patient's survival, GBM remains among the most lethal and resistant malignant tumor [2, 24], and recurrence is nearly universal after a median progression-free survival of 7 to 10 months [25]. Thus, development of new therapies targeting surface molecules or signaling pathways that specifically regulate GBM proliferation or differentiation seems necessary.

In this context, in the present review we summarized the recent evidences demonstrating the participation of mGluR-mediated signaling pathways in GBM proliferation and differentiation, highlighting the putative role of these receptors as new molecular target for management and treatment of this neoplasia.

## GLUTAMATE AS A GROWTH FACTOR FOR GLIOBLASTOMA

**Figure 1:**
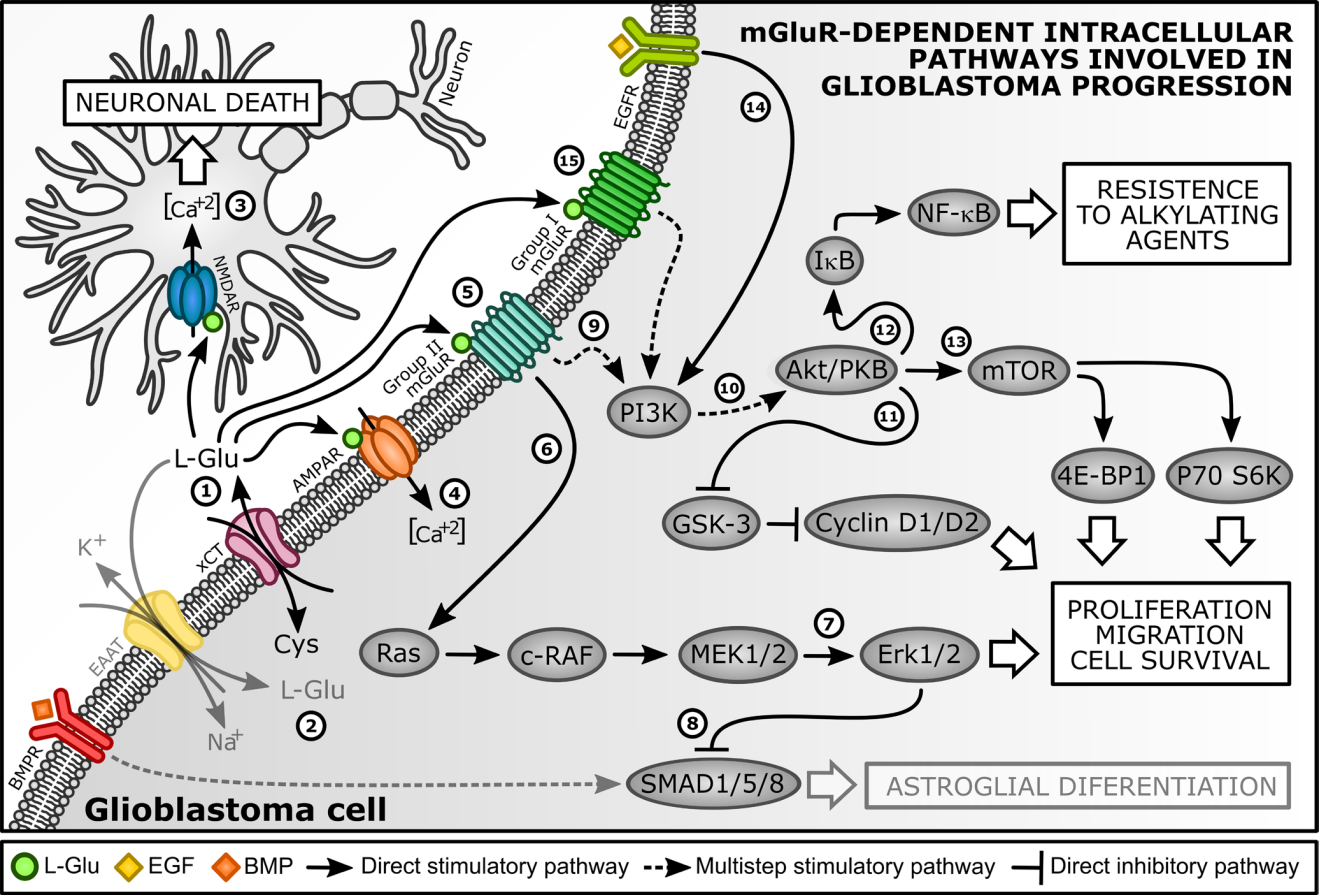
Regulation of GBM proliferative pathways by metabotropic glutamate receptors (mGluR) (1) GBM cells can release low levels of L-Glu mainly by xCT antiporter [27, 28]. (2) Due to loss of EAAT2, GBM cells possessed a low re-uptake rate of L-Glu, maintaining high concentrations of this amino acid in tumor environment [30]. (3) High levels of L-Glu can activate specific NMDAR, which causes neuronal death by excitotoxicity and facilitates tumor bulk expansion [27]. (4) GBM cells expressing mutated Ca^+2^-permeable AMPAR exhibited enhanced migration and proliferation and its blockade led to inhibition of growth and induction of apoptosis [42]. (5) mGluR3 activation by L-Glu induced GBM proliferation and kept these cells under undifferentiated state. In contrast, mGluR3 inhibition eliminated this constraint and promoted astroglial differentiation [68, 73, 76, 78]. (6) Accordingly to Arcella et al. (2005) [73] and Ciceroni et al. (2013) [77], MAPK axis supported mGluR3-induced GBM proliferation, (7) since mGluR3 stimulation increased Erk1/2 phosphorylation and its blockade reduced p-Erk1/2 levels. mGluR3 inhibition plus MEK1/2 blockade showed an additive antiproliferative effect on GBM cells [68]. (8) Moreover, mGluR3-dependet activation of MAPK pathway limited SMAD1/5/8-induced astroglial differentiation, which kept GBM cells under undifferentiated state. Exogenous SMAD1/5/8 stimulation or MEK inhibition prevented this effect [76]. (9) mGluR3 activation stimulated (10) phosphorylation of Akt/PKB *via* PI3K activation and this effect was reversed by receptor inhibition [73, 77]. (11) mGluR3-PI3K axis activation presented a permissive role on GBM cell cycle progression, since mGluR3 inhibition by LY341495 decreased cyclin D1/D2 immunocontent [68, 73], an early marker of G1/S phase transition [79]. (12) mGluR3-PI3K axis was also related to GBM chemoresistance. GSC become sensitive to TMZ, an alkylating agent, only if mGluR3 was inhibited or silenced [77], which was also mimicked by PI3K blockade. NF-κB activation by Akt/PKB limits pro-apoptotic activity of alkylating agents in GBM cells [100]. TMZ increased levels of p-IκB and this effect was reversed by mGluR3 or PI3K blockers. NF-κB bockade enabled TMZ toxicity, occluding permissive action of mGluR3 inhibition, indicating NF-κB lies downstream of Akt/PKB in pathway that restrains TMZ toxicity. (13) Akt/PKB also regulates mTOR, which promotes mRNA translation and protein synthesis through p70 S6K and 4E-BP1 phosphorylation [103]. This signaling pathway was showed to support GBM cells survival [106]. (14) Concomitant activation of EGFR and mGluR3 could act synergistically in GBM aggressiveness, since simultaneous inhibition of these receptors caused maximum apoptosis in GBM cells, as well as reduced their migration. (15) mGluR1 stimulation promoted GBM cells survival through PI3K-Akt/PKB-mTOR pathway activation [41]. mGluR1 inhibition markedly decreased cell viability and inhibited PI3K and Akt/PKB phosphorylation. mGluR1 inhibition also decreased levels of p-mTOR and P70 S6K

Several *in vitro* and *in vivo* studies have demonstrated GBM cells can release high levels of glutamate (L-Glu) to extracellular fluid. Released L-Glu may act as a neurotrophic factor, promoting proliferation and migration of glioma cells as well as contributing to tumor malignancy [26–28]. L-Glu autocrine secretion occurs mainly by cystine-glutamate antiporter (xCT), which exchanges extracellular cystine (Cys) for intracellular L-Glu at a 1:1 stoichiometric ratio [27, 29] (Figure 1, step 1). Moreover, due to loss of excitatory amino acid transporter 2 (EAAT2), GBM cells possess a low re-uptake rate of L-Glu from extracellular fluid, which keeps this aminoacid at a high concentration in extracellular fluid and increases tumor malignancy [27, 30] (Figure 1, step 2). Furthermore, higher levels of L-Glu can trigger a mechanism of neuronal cell death called excitotoxicity [31], which facilitates tumor bulk expansion [27, 32–34] (Figure 1, step 3).

**Figure 2:**
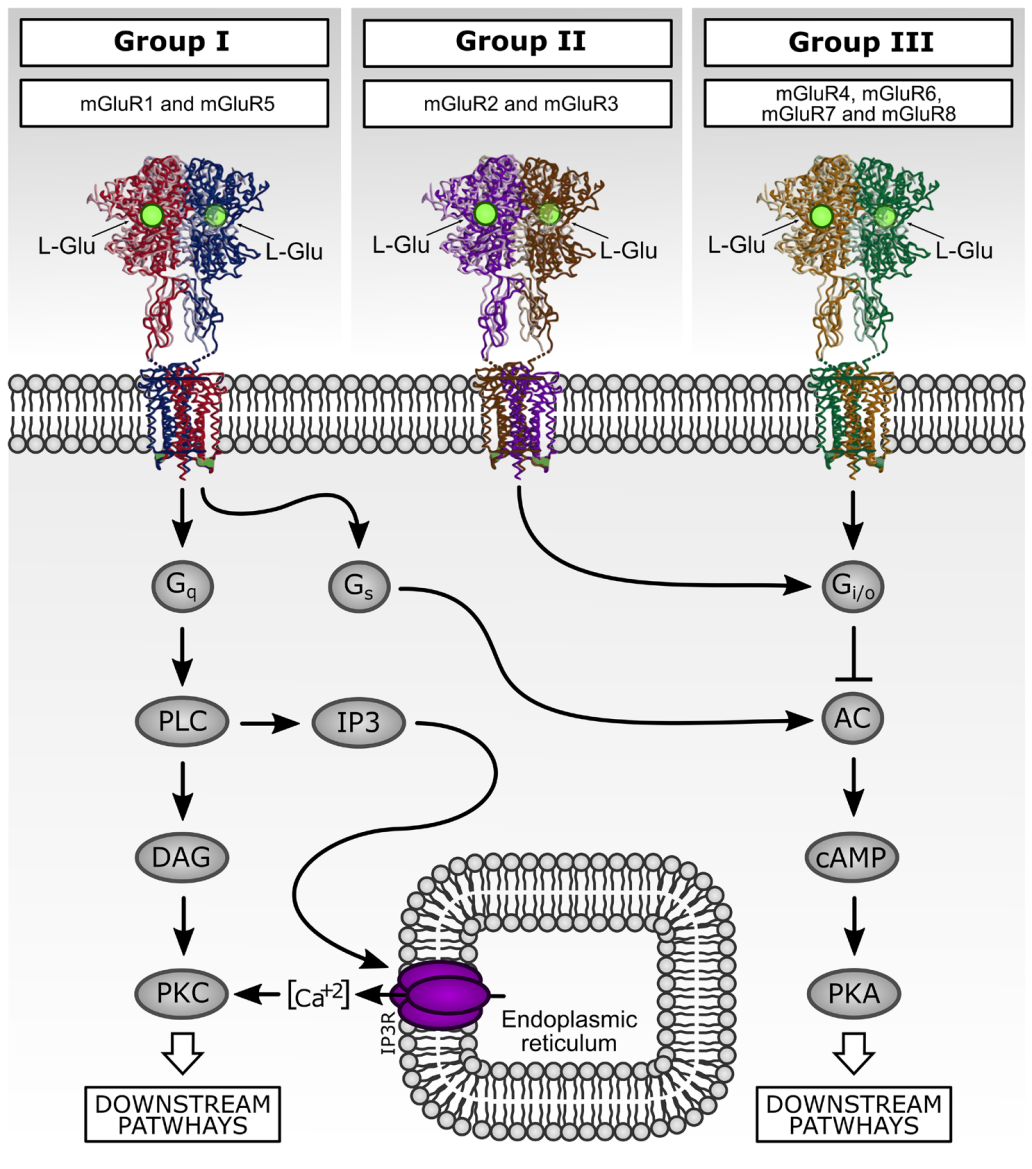
Downstream signaling pathways activated by metabotropic glutamate receptors (mGluR). mGluR family comprises eight subtypes subdivided in three groups according their sequence homology, pharmacology, and second messenger signaling pathway association. Group I mGluR are coupled to G_q_ proteins and their activation stimulates phospholipase C (PLC) and phosphatidylinositol 4,5-biphosphate (PIP_2_) hydrolysis. PIP_2_ hydrolysis generates inositol (1,4,5)-trisphosphate (IP_3_) and diacylglycerol (DAG), which stimulates intracellular Ca^2+^ release from endoplasmic reticulum and activates protein kinase C (PKC), respectively. In contrast, mGluR of group II and III are coupled predominantly to G_i_/_o_ proteins and classically related to inhibition of adenylyl cyclase (AC) and directly regulate ion channel activity and other downstream signaling partners *via* liberation of G_βγ_ subunits.

Extracellular L-Glu activates two classes of membrane receptors: ionotropic glutamate receptors (iGluR: AMPA, NMDA and Kainate receptors), which are ligand-gated ion channels, and metabotropic glutamate receptors (mGluR), which are coupled to G proteins [35, 36]. mGluR family comprises eight subtypes subdivided in three groups according to their sequence homology, pharmacology, and associated-signaling pathway. Group I mGluR are coupled to G_q_ proteins and their activation stimulates phospholipase C (PLC) and phosphatidylinositol 4,5-biphosphate (PIP_2_) hydrolysis. PIP_2_ hydrolysis generates inositol (1,4,5)-trisphosphate (IP_3_) and diacylglycerol (DAG), which stimulates intracellular Ca^2+^ release from endoplasmic reticulum and activates protein kinase C (PKC), respectively [33]. In contrast, mGluR of group II and III are coupled predominantly to G_i_/_o_ proteins, inhibiting adenylyl cyclase (AC) and, thus, decreasing ion channel activity and other downstream signaling pathways [37, 38] (Figure 2). Beyond the well-established role of glutamate receptors in glutamatergic neurotransmission, several evidences are emerging regarding the role of these receptors in cancer biology, especially in malignant brain tumors [39–41].

Activation of a mutated Ca^+2^-permeable form of AMPA receptors (AMPAR) enhanced migration and proliferation of high-grade gliomas (Figure 1, step 4). Blockage of AMPAR by NBQX led to inhibition of glioma growth and induced apoptosis of remaining cells [42, 43]. AMPAR-mediated tumor proliferation seemed to involve a Ca^2+^-dependent activation of Akt/PKB signaling pathway [44], since both NBQX (AMPAR antagonist) and Wortmannin (specific inhibitor of PI3K) reduced the Akt/PKB phosphorylation and decreased the number of tumoral viable cells in culture [45].

Parallel to iGluR, several evidences have demonstrated that mGluR are also functionally important for proliferation and differentiation of distinct types of cancer, including GBM [46]. Group III mGluR is involved in malignancy of a lot of cancers [47]. The implication of mGluR7 in tumor formation has yet to be characterized [46]. mGluR6 gene expression was shown to correlate with higher-grade pediatric CNS tumors [48]. Increased expression of mGluR4 and mGluR8 was reported in human lung adenocarcinoma samples and lung carcinoma cell line and treatment with mGluR8 agonist reduced cell growth and increased apoptosis in this lineage [49]. mGluR4 is overexpressed in more than 40% of malignant melanomas, laryngeal squamous cell carcinomas, and breast carcinomas and its overexpression was correlated with increased mortality in colorectal carcinoma [50]. mGluR4 inhibition suppressed proliferation of mGluR4-expressing colon cancer cell lines [50] whereas mGluR4 activation reduced cell proliferation in medulloblastoma cell lines and inhibits medulloblastoma cell xenografts progression in nude mice [51]. In addition, approximately 77% of human medulloblastoma samples expressed mGluR4, which was inversely correlated with tumor severity, spread, and recurrence [51].

Among Group I mGluR, increased mGluR5 immunoreactivity in human oral squamous cell carcinomas is associated with improved overall survival [52]. mGluR5 antagonist reduced tumor cell migration, invasion, and adhesion in human tongue cancer cells [52] and inhibits cell proliferation of laryngeal cancer [53]. Additionally, mGluR5 overexpression has been shown to induce melanoma development in transgenic mice [54]. Ectopic expression of mGluR1 in normal melanocytes induced melanocyte hyperproliferation *in vitro* and promote melanoma tumor development *in vivo* [55–59]. mGluR1 expression has been widely explored in breast cancer, supporting angiogenesis in these tumors, and silencing of this receptor (GRM1 shRNA) resulted in inhibition of cell proliferation [60]. In addition, mutations and single nucleotide polymorphisms (SNPs) of GRM1 were described in prostate cancer [61] and eight somatic variations of GRM1 were identified in cancers, including lung adenocarcinoma [62]. Group II mGluR is also implicated in a variety of cancer types [47], including melanoma [63]. Both group I and II mGluR are involved in glioma progression and the action *in vitro* and *in vivo* of agonists and antagonists of these receptors will be reported in detail in this review.

The above-mentioned evidences indicate that group I and III mGluR may be considered a potential therapeutic target for both gliomas and other forms of cancer. Moreover, from physiological and pharmacological point of view, there is a growing number of evidences suggesting mGluR are better drug targets than iGluR [64–67]. Compared to iGluR, mGluR play a ‘modulatory’ rather than ‘mediatory’ role in glutamatergic excitatory synaptic transmission [68–71]. Consequently, mGluR ligands (such as agonists or antagonists) might lead to more subtle effects on fast excitatory transmission than iGluR antagonists, which indicates their therapeutic use may be more tolerable for patients [64, 65].

## IN VITRO STUDIES EVALUATING THE ROLE OF mGluR ON GLIOMA PROLIFERATION

Glioma cell cultures have been widely used to elucidate the role of mGluR in cancer malignancy. Some studies have used GBM lineages as cellular model [68, 72–75] while others have used primary cultures from human GBM resections [68, 76–79]. Table 1 (A, B and C) and Table 2 summarizes current literature evaluating mRNA expression and protein immunocontent of mGluR, respectively, in glioma cultures and human glioma resections. Group II mGluR (mGluR2/3) was the most investigated (expressed - mRNA - and immunodetected - protein) in the majority of human glioma biopsy samples, primary cultures, and glioma lineages. In order to clarify the role of mGluR on proliferation, invasiveness, and migration, several assays were performed treating GBM cells with antagonists and/or agonists of these receptors.

Table 1A: RNA expression of group I mGluR subtypes in cellular malignant glioma models.

**Table:** 

Metabotropic glutamate receptors		mRNA			
		Sample		Expression	Reference
Group I	mGluR1	Human Glioma Lineage	U-87 MG	Yes	[72]
				No	[73]
			U-343	Yes	[72]
			MOGGCCM	Yes	[72]
		Resection	AZ21 (Low grade astrocytoma)	Yes	[48]
			AZ8 (Low grade astrocytoma)	Yes	[48]
			AZ7 (Low grade astrocytoma)	Yes	[48]
			AZ6 (Low grade astrocytoma)	Yes	[48]
			AZ5 (Low grade astrocytoma)	Yes	[48]
			AZ4 (Low grade astrocytoma)	Yes	[48]
			AZ3 (Low grade astrocytoma)	No	[48]
			AZ2 (Low grade astrocytoma)	Yes	[48]
			GB4 (Glioblastoma)	Yes	[48]
			GB3 (Glioblastoma)	Yes	[48]
			GB2 (Glioblastoma)	Yes	[48]
			GB1 (Glioblastoma)	No	[48]
	mGluR5	Human Glioma Lineage	U-87 MG	Yes	[72]
				No	[73, 74]
			U-343	Yes	[72]
			MOGGCCM	Yes	[72]
			U-178 MG	Yes	[74]
			U-251	No	[68]
		Primary culture from human glioma	FCN-9	No	[68]
			MZC-12	No	[68]
			CDR-97	No	[68]
		Resection	Anaplastic astrocytoma (52 years old)	No	[79]
			Astrocytoma grade II (12 years old)	Yes	[79]
			Glioblastoma (53 years old)	No	[79]
			Glioblastoma (68 years old)	No	[79]
			Glioblastoma (82 years old)	No	[79]
			Glioblastoma (58 years old)	No	[79]
			Glioblastoma (55 years old)	No	[79]
			Astrocytoma grade I (38 years old)	No	[79]
			AZ21 (Low grade astrocytoma)	Yes	[48]
			AZ8 (Low grade astrocytoma)	No	[48]
			AZ7 (Low grade astrocytoma)	Yes	[48]
			AZ6 (Low grade astrocytoma)	Yes	[48]
			AZ5 (Low grade astrocytoma)	Yes	[48]
			AZ4 (Low grade astrocytoma)	No	[48]
			AZ3 (Low grade astrocytoma)	Yes	[48]
			AZ2 (Low grade astrocytoma)	Yes	[48]
			GB4 (Glioblastoma)	Yes	[48]
			GB3 (Glioblastoma)	Yes	[48]
			GB2 (Glioblastoma)	Yes	[48]
			GB1 (Glioblastoma)	Yes	[48]

Table 1B: RNA expression of group II mGluR subtypes in cellular malignant glioma models.

**Table:** 

**Metabotropic glutamate receptors**		**mRNA**			
		**Sample**		**Expression**	**Reference**
Group II	mGluR2	Human Glioma Lineage	U-87 MG	Yes	[72, 73]
			U-343	Yes	[72]
			MOGGCCM	Yes	[72]
			U-251	Yes	[68]
		Primary culture from human glioma	FCN-9	No	[68]
			MZC-12 and MSS-5	Yes	[68]
			FLS-10	No	[68]
			LTN-12	No	[68]
			BRT-3	No	[68]
			CRL-8	No	[68]
			CDR-97	No	[68]
			Glioma Stem Cells	No	[76–78]
		Resection	Glioblastoma	Yes	[78]
			AZ21 (Low grade astrocytoma)	Yes	[48]
			AZ8 (Low grade astrocytoma)	Yes	[48]
			AZ7 (Low grade astrocytoma)	Yes	[48]
			AZ6 (Low grade astrocytoma)	Yes	[48]
			AZ5 (Low grade astrocytoma)	Yes	[48]
			AZ4 (Low grade astrocytoma)	Yes	[48]
			AZ3 (Low grade astrocytoma)	Yes	[48]
			AZ2 (Low grade astrocytoma)	Yes	[48]
			GB1, GB2, GB3 and GB4 (Glioblastomas)	Yes	[48]
	mGluR3	Human Glioma Lineage	U-87 MG	Yes	[72, 73]
			U-343	No	[72]
			MOGGCCM	Yes	[72]
			U-251	Yes	[68]
		Primary culture from human glioma	FCN-9	Yes	[68]
			MZC-12	Yes	[68]
			FLS-10, LTN-12 and CDR-97	No	[68]
			MSS-5	Yes	[68]
			BRT-3	Yes	[68]
			CRL-8	Yes	[68]
			Glioma Stem Cells	Yes	[76–78]
		Resection	Glioblastoma	Yes	[78]
			AZ21 (Low grade astrocytoma)	Yes	[48]
			AZ8 (Low grade astrocytoma)	Yes	[48]
			AZ7 (Low grade astrocytoma)	Yes	[48]
			AZ6 (Low grade astrocytoma)	Yes	[48]
			AZ5 (Low grade astrocytoma)	Yes	[48]
			AZ4 (Low grade astrocytoma)	Yes	[48]
			AZ3 (Low grade astrocytoma)	Yes	[48]
			AZ2 (Low grade astrocytoma)	Yes	[48]
			GB1, GB3 and GB4 (Glioblastomas)	Yes	[48]
			GB2 (Glioblastoma)	No	[48]
			U-343	Yes	[72]
			MOGGCCM	Yes	[72]
		Resection	AZ21 (Low grade astrocytoma)	Yes	[48]
			AZ8 (Low grade astrocytoma)	No	[48]
			AZ7 (Low grade astrocytoma)	Yes	[48]
			AZ6 (Low grade astrocytoma)	Yes	[48]
			AZ5 (Low grade astrocytoma)	Yes	[48]
			AZ4 (Low grade astrocytoma)	Yes	[48]
			AZ3 (Low grade astrocytoma)	Yes	[48]
			AZ2 (Low grade astrocytoma)	Yes	[48]
			GB1, GB3 and GB4 (Glioblastomas)	Yes	[48]
			GB2 (Glioblastoma)	No	[48]

Table 1C: RNA expression of group III mGluR subtypes in cellular malignant glioma models.

**Table:** 

**Metabotropic glutamate receptors**		**mRNA**			
		**Sample**		**Expression**	**Reference**
Group III	mGluR4	Human Glioma Lineage	U-87 MG	No	[72, 73]
			U-343	Yes	[72]
			MOGGCCM	Yes	[72]
		Resection	AZ21 (Low grade astrocytoma)	Yes	[48]
			AZ8 (Low grade astrocytoma)	Yes	[48]
			AZ7 (Low grade astrocytoma)	Yes	[48]
			AZ6 (Low grade astrocytoma)	No	[48]
			AZ5 (Low grade astrocytoma)	Yes	[48]
			AZ4 (Low grade astrocytoma)	Yes	[48]
			AZ3 (Low grade astrocytoma)	No	[48]
			AZ2 (Low grade astrocytoma)	No	[48]
			GB1, GB2, GB3 and GB4 (Glioblastoma)	Yes	[48]
	mGluR6	Human Glioma Lineage	U-87 MG	Yes	[72]
			U-343	Yes	[72]
			MOGGCCM	Yes	[72]
		Resection	AZ21 (Low grade astrocytoma)	Yes	[48]
			AZ8 (Low grade astrocytoma)	No	[48]
			AZ7 (Low grade astrocytoma)	No	[48]
			AZ6 (Low grade astrocytoma)	No	[48]
			AZ5 (Low grade astrocytoma)	No	[48]
			AZ4 (Low grade astrocytoma)	No	[48]
			AZ3 (Low grade astrocytoma)	No	[48]
			AZ2 (Low grade astrocytoma)	No	[48]
			GB4 (Glioblastoma)	Yes	[48]
			GB3 (Glioblastoma)	Yes	[48]
			GB2 (Glioblastoma)	Yes	[48]
			GB1 (Glioblastoma)	No	[48]
	mGluR7	Human Glioma Lineage	U-87 MG	Yes	[72]
				No	[73]
			U-343	Yes	[72]
			MOGGCCM	Yes	[72]
		Resection	AZ21 (Low grade astrocytoma)	Yes	[48]
			AZ8 (Low grade astrocytoma)	Yes	[48]
			AZ7 (Low grade astrocytoma)	Yes	[48]
			AZ6 (Low grade astrocytoma)	Yes	[48]
			AZ5 (Low grade astrocytoma)	Yes	[48]
			AZ4 (Low grade astrocytoma)	No	[48]
			AZ3 (Low grade astrocytoma)	Yes	[48]
			AZ2 (Low grade astrocytoma)	Yes	[48]
			GB1, GB2, GB3 and GB4 (Glioblastoma)	Yes	[48]
	mGluR8	Human Glioma Lineage	U-87 MG	Yes	[72]
			U-343	Yes	[72]
			MOGGCCM	Yes	[72]
		Resection	AZ21 (Low grade astrocytoma)	Yes	[48]
			AZ8 (Low grade astrocytoma)	No	[48]
			AZ7 (Low grade astrocytoma)	Yes	[48]
			AZ6 (Low grade astrocytoma)	Yes	[48]
			AZ5 (Low grade astrocytoma)	Yes	[48]
			AZ4 (Low grade astrocytoma)	Yes	[48]
			AZ3 (Low grade astrocytoma)	Yes	[48]
			AZ2 (Low grade astrocytoma)	Yes	[48]
			GB1, GB3 and GB4 (Glioblastoma)	Yes	[48]
			GB2 (Glioblastoma)	No	[48]

**Table 2:** Protein immunocontent of mGluR subtypes in cellular malignant glioma models

**Metabotropic glutamate receptors**		**Protein (Western Blot)**			
		**Sample**		**Expression**	**Reference**
Group I	mGluR1	Human Glioma Lineage	U-87 MG	Yes	[41]
			U-373	No	[75]
		Resection	Astrocytoma II	No	[75]
			Astrocytoma Anaplastic	No	[75]
			Glioblastoma	No	[75]
	mGluR5	Human Glioma Lineage	U-87 MG	Yes	[41, 75]
			U-373	Yes	[75]
			U-118	Yes	[75]
		Resection	Astrocytoma II	Yes	[75]
			Astrocytoma Anaplastic	Yes	[75]
			Glioblastoma	Yes	[75]
Group II	mGluR2/3	Human Glioma Lineage	U-87 MG	Yes	[68]
			U-373	No	[68]
			A172	Yes	[68]
		Primary culture from human glioma	FCN-9	Yes	[68]
			MZC-12	Yes	[68]
			MSS-5	Yes	[68]
			BRT-3	Yes	[68]
			CRL-8	Yes	[68]
			GSS 98	Yes	[68]
			DMD 126	Yes	[68]
			MTR4	Yes	[68]
			Glioma Stem Cells	Yes	[76, 77]
		Resection	Astrocytoma II	Yes	[75]
			Astrocytoma Anaplastic	Yes	[75]
			Glioblastoma	Yes	[75]
	mGluR2	Primary culture from human glioma	Glioma Stem Cells	No	[78]
		Resection	Glioblastoma	Yes	[78]
	mGluR3	Primary culture from human glioma	Glioma Stem Cells	Yes	[78]
		Resection	Glioblastoma	Yes	[78]

Arcella et al. (2005) showed pharmacological blockade of mGluR2/3 induced antiproliferative effects in U-87 MG glioma cell line. Daily addition (four days) of mGluR2/3 antagonists (LY 341495 - 1 μM; MTPG - 100 μM; or EGLU - 100 μM) to U-87 MG cultures reduced cell proliferation in a time-dependent manner, while did not induce apoptosis [73] (Figure 1, step 5). However, this treatment altered cell cycle, since FACS analysis showed antagonist reduced percentage of cells in S and G2M phases after two but not after four days of exposure. Furthermore, in cultures deprived of serum by 72 h, LY 341495 treatment reduced EGF-induced cyclin D1/D2 protein expression (early marker of the G1/S phase transition [79]).

In another study, using primary cultures from human GBM biopsies, D’Onofrio et al. (2003) showed pharmacological blockade of mGluR3 reduced cell proliferation [68]. This effect was observed in all selected cultures mGluR3^+^. Application of antagonist LY 341495 (1 μM) to growing medium once a day (for four days) reduced linear phase of growth in mGluR3+ cultures, with cell number being substantially reduced at 4^th^ day of treatment. Cell growth was restored two days after washing out LY 341495, indicating that antiproliferative effect was reversible (*i.e*., antagonist was cytostatic, not cytotoxic). For excluding involvement of other mGluR, authors performed an additional experiment using lower concentrations of LY 341495 and this antagonist was able to reduce linearly cell growth at concentrations of 1 and 10 nM, indicating that inhibition of mGluR3 was responsible for antiproliferative effect of LY 341495. This result was further corroborated by evidence that EGLU (100 μM), another antagonist of mGluR3, mimicked LY 341495 action on cell growth.

Glioma stem cells (GSC) are brain tumor-initiating undifferentiated cells [80], which have multipotential differentiation capacity, high tumorigenic potential and low proliferation rate [81, 82]. Normally, GSC are obtained from adult human GBM biopsy samples and form in culture classical floating aggregates, named tumor spheres. These cells are very chemoresistant and radioresistant and therefore probably responsible for tumor progression and recurrence after conventional GBM resection [82]. Ciceroni et al. (2008) and Zhou et al. (2014) observed GSC express mGluR3 protein (but not mGluR2) and its pharmacological blockade promoted an astroglial differentiation of GSC [76, 78]. Zhou et al. (2014) showed mGluR3 blockade by LY 341495 (100 nM) for 48 h resulted in a decreased proliferation combined with an increase in GSC GFAP^+^ cells (classical marker for mature astrocytes) and a decrease of GSC nestin^+^ cells (classical marker for neural stem cells) [78]. In addition, stimulation of mGluR3 for 48 h by specific agonist LY 379268 (100 nM) has no effect on GSC proliferation and differentiation, which suggests mGluR3 activity is necessary to maintain proliferation but is incapable of stimulating it *per se* (Figure 1, step 5).

Ciceroni et al. (2008) showed treatment of GSC with antagonist LY 341495 (100 nM) promoted astrocytic-like differentiation of cells (increasing GFAP^+^ cells) and nestin^+^ cells were virtually absent after 14 days of treatment [76]. Treatment with agonist LY 379268 (100 nM) did not affect differentiation of cells. In another set of experiments, these authors cultured GSC under differentiating conditions (medium deprived of mitogens and containing 10% of fetal calf serum) for 8 days and received treatments with mGluR3 ligants. After, cells were enzymatically dissociated, transferred to uncoated 96-well plates, and regrown under proliferating conditions without any mGluR3 ligants (medium containing epidermal growth factor - EGF - plus basic fibroblast growth factor - bFGF - and lacking serum) for plus 12 days. Treatment of cells with agonist LY 379268 during differentiation phase led to formation of tumor spheres in proliferative phase. In contrast, cells treated with antagonist LY 341495 originated cultures containing exclusively adherent astrocyte-like cells. This indicated that treatment with mGluR3 antagonist was sufficient to maintain GSC towards astroglial differentiation even under conditions in which they normally proliferate and maintain their undifferentiated state (Figure 1, step 5).

For both above-mentioned studies [76, 78], GSC did not respond to agonist LY 379268 probably because mGluR3 were constantly stimulated by L-Glu already present in culture medium. Concentrations of extracellular L-Glu in GSC cultured for 8 and 14 days under differentiating conditions (medium deprived of mitogens and containing serum) were about 60 μM and 70 μM, respectively [76]. These concentrations exceed the reported EC_50_ value for L-Glu and recombinant mGluR3 by 5-10 fold [83], which is sufficient to saturate all mGluR3.These results point to the notion that activation of mGluR3 by endogenous L-Glu allows proliferation of GSC by limiting astroglial differentiation and that receptor blockage eliminates this constraint, thereby promoting cell differentiation. This hypothesis is supported by work performed by Yelskaya et al. (2013), in which U-87 MG cultures where treated with Riluzole (1-100 μM), a drug that blocks the secretion of L-Glu and enhances its uptake from extracellular space [47]. Riluzole inhibited proliferation of cells in a dose-dependent manner, suggesting that absence of L-Glu in extracellular medium prevents glutamate-dependent proliferation and putative activation of mGluR3 in U-87 MG cells [84].

Group II mGluR are known to be able to activate the MAPK and PI3K pathways [37, 85–89], which are usually activated in response to proliferating agents [90, 91]. Arcella et al. (2005) [73] showed treatment of U-87 MG cultures with antagonist LY 341495 (1 μM) reduced activation of MAPK (assessed by WB analysis of p-Erk1/2) and PI3K (assessed by WB analysis of p-Akt/PKB) pathways. All of these effects were reversed by addition of agonist LY 379268 (1 μM), which was inactive *per se*. WB analysis also showed exposure to LY 341495 did not alter mGluR2/3 immunocontent (at least up to four days of treatment). Another study indicated that activation of mGluR3 could have a permissive role on stimulation of MAPK and PI3K pathways in GSCs dissociated cultures [77]. GSCs dissociated from tumor spheres were starved from mitogens and then treated with agonist LY 379268 (100 nM), which inhibited forskolin-stimulated cyclic adenosine monophosphate (cAMP) formation and increased p-Erk1/2 and p-Akt/PKB levels. All these effects were reversed by antagonist LY341495 (100 nM). In addition, treatment with LY 341495 also reversed EGF- and bFGF-induced increase in p-Erk1/2 and p-Akt/PKB levels. LY 341495 alone did not affect EGF receptor (EGFR) autophosphorylation in response to EGF, which suggests that this drug had no direct effects on EGFR (Figure 1, step 6, 7, 9 and 10).

D’Onofrio et al (2003) examined the immunocontent of cyclin D1 and D2 and the activation of MAPK pathway in primary GBM cell cultures in serum-deprived conditions and in proliferative conditions (cells incubated with EGF for 8 h, for assessment of cyclin D1/D2, or for 10 min, for assessment of MAPK pathway) [68]. Addition of 1 μM of mGluR3 antagonist LY 341495 (in combination with EGF) reduced EGF-induced increase in immunocontent of both cyclin D1/D2 and p-Erk1/2. Effect on cyclin D1/D2 protein expression was partially reversed by agonist LY 379268 (1 μM) (Figure 1, step 11). To assess whether inhibition of MAPK pathway was the only mechanism responsible for antiproliferative effect of LY 341495, authors studied association of this drug with MAPK kinase (MEK) inhibitor, PD 98059. Both LY 341495 (1 μM) and PD 98059 (30 μM) reduced cell number in about 45%. Ability of LY 341495 and PD 98059 to reduce Erk1/2 phosphorylation suggests mGluR2/3-MAPK axis supports proliferation rate of human GBM cells. Since anti-proliferative effect of LY 341495 was not totally obliterated by PD 98059, other proliferation pathways may be also being controlled by mGluR2/3 in GBM cells (Figure 1, step 6 and 7).

Ciceroni et al. (2008) focused on interaction between mGluR3 and bone morphogenetic proteins (BMP), which are known to promote astroglial differentiation of GSC [92]. BMP bind to membrane receptors of BMP/TGF-Δ/activin family, which leads to phosphorylation and translocation of Smad1/5/8 proteins to nucleus [93]. Addition of exogenous BMP4 (100 ng/mL) plus mGluR3 agonist LY 379268 (100 nM) prevented BMP4-induced nuclear translocation and phosphorylation of Smad. Inhibitory action of LY 379268 was unaffected by non-metabolizable cAMP analogue, 8-Bromo-cAMP (1 mM), but was prevented by MAPK kinase (MEK) inhibitor, UO126 (30 μM), which was inactive *per se*. Moreover, cultures exposed to mGluR3 antagonist LY 341495 (100 nM) enhanced Smad1/5/8 phosphorylation to an extent similar to BMP4. These results suggested that activation of mGluR3 could inhibit BMP4 receptor signaling by activation of MAPK pathway. In addition, activation of mGluR3-MAPK pathway by endogenous L-Glu presented in medium may limit BMP4-induced differentiating activity, thus contributing to support undifferentiated state of GSCs, and eventually GBM growth and relapse [76] (Figure 1, step 8).

Yelskaya et al. (2013) reported that a combination of mGluR2/3 antagonist LY 341495 and Gefitinib, an EGFR inhibitor, works most efficiently to inhibit proliferation and migration of U-87 MG cells and induced apoptosis in this cell lineage [84]. They also investigated the efficacy of different classes of drugs (AMPAR antagonist - NBQX; mGluR2/3 antagonist - LY341495; EGFR inhibitor - Gefitinib; and PI3K inhibitors - Wortmannin and PI 828) in inhibiting proliferation of U-87 MG cells (24 h). A combination of Gefitinib (25 μM) with LY 341495 (1 μM) or PI 828 (2 μM) was more effective to inhibit proliferation of U-87 MG cells when compared to individual drugs alone. Using TUNEL assay, authors showed treatment with Gefitinib resulted in increased apoptosis compared to control group. Gefitinib in combination with PI 828 or Wortmannin (5 μM) did not increase apoptosis in cell cultures. However, treatment with Gefitinib plus NBQX (5 μM) or Gefitinib plus LY 341495 increased apoptosis compared to Gefitinib, NBQX or LY 341495 alone. Maximum percentage of apoptosis was observed in treatment with Gefitinib plus LY341495. Distance migrated by cells (wound healing assay) was significantly reduced with Gefitinib plus LY 341495 treatment when compared to treatments with Gefitinib or LY 341495 alone or control group (Figure 1, step 14).

An interesting question that arises from above-mentioned works is whether mGluR3 antagonists could interact with classical chemotherapies and whether activation of this receptor in malignant gliomas could control expression of proteins implicated in chemoresistance. In this context, Ciceroni et al. (2013) showed mGluR3 inhibition enables cytotoxic action of TMZ in GSC cultures [77]. TMZ (250 μM) did not affect cell viability when applied alone, but became toxic when combined with LY 341495 (100 nM) and LY 2389575 (100 nM), two mGluR3 antagonists. mGluR3 agonist LY 379268 (100 nM) was inactive *per se*, but reversed the permissive action of LY 341495 on TMZ toxicity. siRNA-induced knockdown of mGluR3 also enabled TMZ toxicity and antagonist LY 341495 did not further amplify TMZ toxicity in mGluR3 silenced cells [77]. This data suggests that a possible activation of mGluR3 by GSC-autocrine release of L-Glu could restrain toxic action of TMZ, increasing chemoresistance of these tumor cells (Figure 1, step 12). GSC were treated with other anticancer drugs (etoposide, irinotecan, irinotecan metabolite - SN 38, cisplatin or paclitaxel) alone or combined with mGluR3 antagonist LY341495 and these treatments had no significant effect on GSC viability, suggesting mGluR3 receptors selectively control responses of cells to TMZ and could not be extended to other chemotherapeutic agents.

In order to evaluate mechanisms underlying mGluR3-induced chemoresistance, Ciceroni et al. (2013) treated GSC with molecules that interfere in three major signaling pathways activated by mGluR3 [77]: inhibition of adenylyl cyclase (AC) activity, activation of MAPK pathway and activation of PI3K pathway [85, 88, 94, 95]. Cell permeable cAMP analog, 8-Bromo-cAMP (1 mM), did not affect synergism between mGluR3 blockade and TMZ. MAPK kinase (MEK) inhibitor, UO126 (30 μM), had mild effect on TMZ toxicity. In contrast, PI3K inhibitor LY 294002 (10 μM) had a permissive action on TMZ toxicity, mimicking the effect of mGluR3 blockade. Actions of LY 294002 and LY 341495 were less than addictive, suggesting mGluR3 inhibition facilitates cytotoxicity by limiting activation of PI3K pathway. This hypothesis was supported by the use of GSCs expressing a constitutively active form of t PI3K substrate, Akt/PKB [96]. In these cells, in which PI3K pathway was active in spite of mGluR3 blockade, synergism between LY 341495 and TMZ was largely attenuated (Figure 1, step 12).

The sensitivity of cancer cell lineages to various inhibitors and chemotherapeutic drugs is often associated with genetic mutations of key elements in the Ras-Raf-MEK-ERK and PI3K-PTEN-Akt/PKB-mTOR pathways [97, 98]. Akt/PKB is known to activate nuclear factor-κB (NF-κB) by phosphorylating IκB kinase [99], which limits the pro-apoptotic activity of DNA-alkylating agents in glioma cells [100]. In Ciceroni et al. (2013) study, treatment with TMZ activated NF-κB, showed by increased levels of IκB phosphorylation. This effect was reversed by mGluR3 antagonist, LY 341495, or by PI3K inhibitor, LY 294002. The specific NF-κB inhibitor JSH-23 (10 μM) [101] enabled TMZ toxicity and occluded permissive action of LY 341495 in GSCs. Similar effects were obtained with salicylic acid, which also inhibits NF-κB [102]. As opposed to LY 341495, JSH-23 could still enhance TMZ toxicity in GSCs expressing the constitutively active form of Akt/PKB, indicating NF-κB lies downstream to Akt/PKB in pathway that restrains TMZ toxicity (Figure 1, step 12). Akt/PKB also regulates mammalian target of rapamycin (mTOR), which promotes mRNA translation and protein synthesis by phosphorylating p70 S6K and 4E-BP1 [103]. mGluR stimulation activates mTOR pathway [104, 105] and inhibitors of Akt/PKB-mTOR pathway are under development for treatment of cancer, including malignant gliomas [106, 107]. In Ciceroni et al. (2013) work, it was shown selective mTOR inhibitor rapamycin (10 nM) did not mimic, but rather abolished permissive action of mGluR3 blockade on TMZ toxicity (Figure 1, step 13).

Clinical efficacy of TMZ is limited by DNA-repairing enzyme, O6-methylguanine-DNA methyltransferase (MGMT), which removes DNA adducts generated by alkylating agents [108]. In Ciceroni et al. (2013) [77] study, GSC clones constitutively expressed MGMT and treatment with TMZ alone increased MGMT mRNA levels 3 h after its application and slightly reduced MGMT protein levels at 24 and 48 h. When TMZ was combined with LY 341495, MGMT mRNA did not increase and MGMT protein levels were markedly reduced. Moreover, action of LY341495 was mimicked by siRNA-induced knockdown of mGluR3, or PI3K inhibitor (LY 294002), or by NF-κB inhibitor (JSH-23). Finally, the permissive action of LY 341495, LY 294002, or JSH-23 was no longer seen in GSC overexpressing MGMT, demonstrating that synergistic action of mGluR3 blockade and TMZ treatment was mediated by inhibition of MGMT expression. This hypothesis was supported by evidence that treatment with MGMT inhibitor, O6-benzylguanine (10 μM), enabled TMZ toxicity. These results suggest that mGluR3-dependent TMZ toxicity restrains is supported by induction of MGMT transcription *via* an intracellular signaling pathway that sequentially involves PI3K and NF-κB.

A recent study described, for the first time, an anti-cancer role of mGluR1 in gliomas [41]. Zhang et al. (2015) showed treatment with selective mGluR1 antagonist Bay 36-7620 (50μM) or Riluzole (50μM), a glutamate release inhibitor approved for amyotrophic lateral sclerosis [47, 109], markedly decreased cell viability and increased LDH release in U-87 MG lineage. These treatments and mGluR1 knockdown (using siRNA technology) significantly increased apoptotic rate in these glioma cells. In addition, Riluzole or Bay 36-7620 increased immunocontent of cleaved PARP and caspase-3, whereas immunocontent of pro-PARP and pro-caspase-3 was not altered. Similar results were observed after mGluR1 knockdown. Authors also tested whether treatment with Riluzole or Bay 36-7620 and transfection with mGluR1 targeted siRNA could induce inhibition of PI3K activity in U87 cells. U-87 MG cells showed a significantly decreased phosphorylation of PI3K after mGluR1 blockade as well as after mGluR1 targeted siRNA. mGluR1-dependent inhibition of PI3K resulted in inhibition of Akt/PKB phosphorylation at both Ser473 and Thr308 residues. Moreover, mGluR1 inhibition decreased expression levels of p-mTOR and P70 S6K, one of the best-characterized targets of mTOR complex. In contrast, expression of PTEN was not changed by antagonists or siRNA transfection. All these data provided strong evidence that activation of PI3K-Akt/PKB-mTOR pathway was suppressed after mGluR1 inhibition (Figure 1, step 15).

The majority of above-mentioned *in vitro* works indicates that endogenous activation of mGluR1 and mGluR3 increase the proliferation of GBM cells and that MAPK and PI3K pathways may be involved in this process. All of these studies are particularly interesting from a therapeutic standpoint since ligands of these types of mGluR represent an expanding class of drugs endowed with high receptor affinity, elevated brain penetration, and good profile of safety and tolerability [65, 83]. These features make these drugs potential candidates for *in vivo* studies evaluating progression and aggressiveness of malignant brain tumors.

## IN VIVO STUDIES EVALUATING THE ROLE OF mGluR ON GLIOMA TUMOR PROGRESSION

Arcella et al. (2005) [73] have shown that a continuous systemic infusion of mGluR2/3 antagonist LY 341495 reduced growth of GBM cells in two independent *in vivo* models. U-87 MG cells were implanted under skin (2 × 10^6^ cells/mL) of mice, which were subcutaneously implanted with osmotic minipumps releasing saline, LY 341495 (1 mg/kg per day), EGLU (1 mg/kg per day), LY 379268 (1 mg/kg per day), or LY 341495 plus LY 379268 during 28 days. Analysis of tumor weight showed chronic infusion of antagonists LY 341495 or EGLU reduced GBM growth. On the other hand, infusion of mGluR2/3 agonist LY 379268 did not affect tumor growth and failed to fully reverse LY341495 effect. These results corroborate with Zhou et al. (2014) *in vitro* study [78], in which activation of mGluR2/3-dependent signaling pathway is necessary to maintain tumor growth but is incapable of stimulating it *per se*.

In another set of experiments, Arcella et al. (2005) implanted U-87 MG cells into brain left caudate nucleus of nude mice and immunohistochemical analysis revealed that these cells showed a higher expression of mGluR2/3 and Ki-67 (a cellular marker for proliferation) [73]. After magnetic resonance imaging (MRI) analysis (7^th^ day after cell implantation), selected mice with similar tumor sizes were subcutaneously implanted with osmotic minipumps releasing either saline or antagonist LY 341495 (10 mg/kg per day). Treatment during 7 days with LY 341495 reduced tumor size and drug effect was particularly evident during exponential phase of tumor growth (between the 21^st^ and 28^th^ days after cell implantation). Withdrawal of LY 341495 on 21^st^ day allowed growing of tumor to the same extent as control group, suggesting effect of LY 341495 was reversible and cytostatic. Treatment with LY 341495 also reduced the number of Ki-67^+^ cells in tumor specimens.

**Figure 3:**
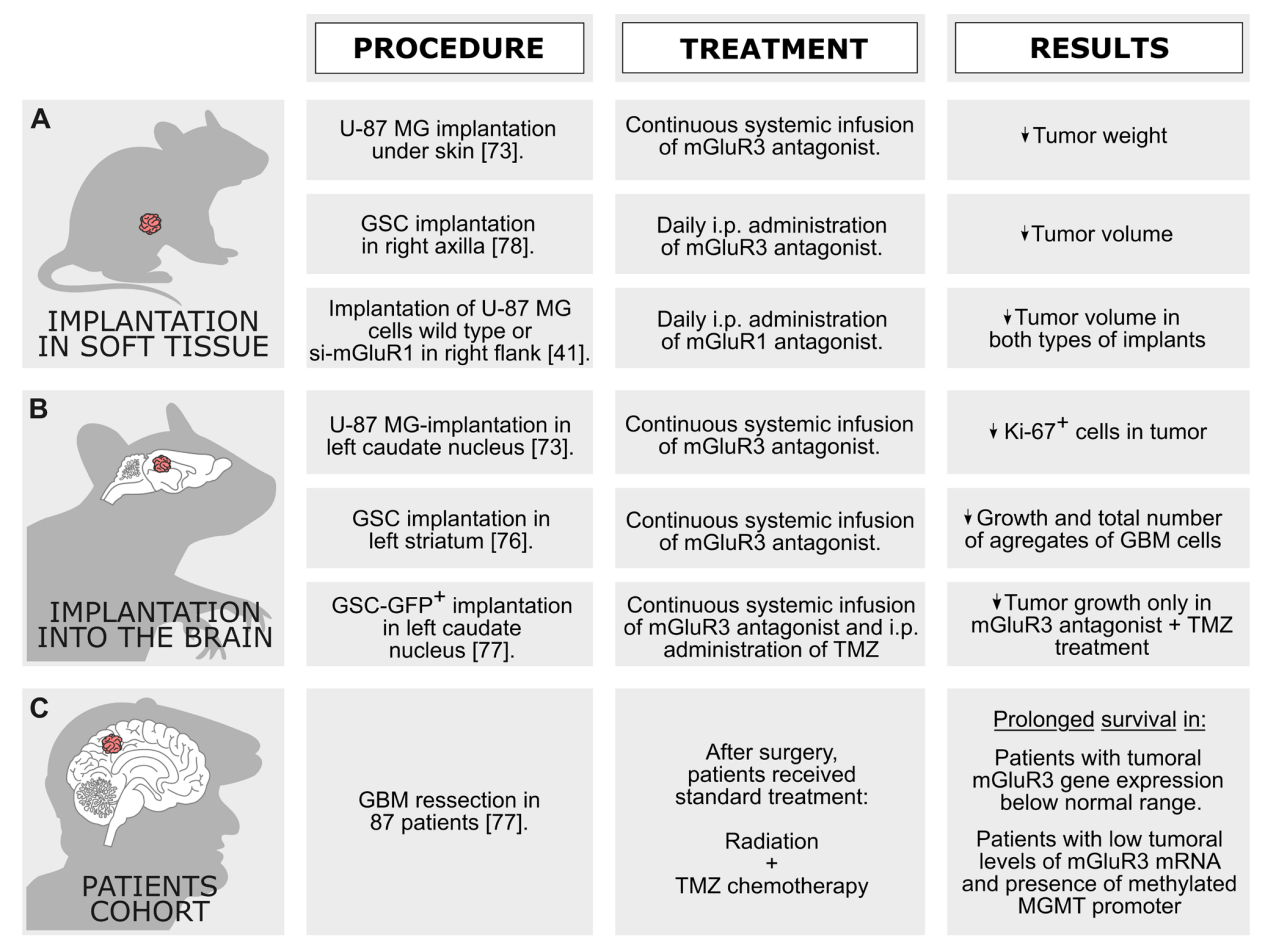
***In vivo*** models employed to study the role of mGluR on brain tumor growth and aggressiveness. **A**. and **B**. Effects of mGluR ligands in two mice xenograft models. **C**. Relation between tumoral mGluR3 mRNA expression and GBM patients’ survival.

These two xenograft models above-described may be complementary (Figure 3A and 3B). Growth of implanted cells in a soft tissue (*i.e*., subcutaneously) evaluates mainly proliferation rate of tumor on an adequate energy supply. On the other hand, growth of glioma cells in brain requires multiple processes, such as excitotoxic-mediated neuronal death, expression of enzymes that degrade extracellular matrix, and expression of ion channels that drive movement of water out of cell [110]. Unfortunately, work performed by Arcella et al. (2005) have no information regarding outcome of mice treated with LY 341495, since animals were not allowed to survive beyond the 4^th^ week of tumor growth. Nevertheless, authors said none of the five mice died during the four weeks of observation in group treated with this antagonist [73].

Ciceroni et al. (2008) showed continuous pharmacological blockade of mGluR3 reduced growth of infiltrating brain tumors originating from GSC xenografts [76]. GSC spheres were suspended in their growing medium and then infused into left striatum of nude mice (3 × 10^5^ cells/3 μL). Immediately after cell transplantation, mice were subcutaneously implanted with osmotic minipumps releasing mGluR3 agonist LY 379268 or antagonist LY 341495 (both at a rate of 1 mg/kg per day during 3 months), or filled with saline solution. MRI analysis carried out at 3 months showed signal alterations in brain parenchyma of control and LY 379268 treated mice, however, no changes were observed in mice treated with antagonist LY 341495. Histological analysis revealed presence of small and large aggregates of GBM cells in brain parenchyma, which were characterized by nuclear atypia and high mitotic activity. These features are consistent with primitive advanced-stage GBM, where tumors migrate and disseminate asymmetrically along blood vessels and fiber tracts and not grow uniformly [111]. Cell aggregates were consistently found in ipsilateral neostriatum, as well as along the intra- and inter-hemispheric white matter in both control and LY 379268 treated mice. On the other hand, aggregates were absent or present to a very low extent in animals treated with antagonist LY 341495, which indicates that mGluR3 blockage inhibited GSC-dependent generation of tumor cell progeny and/or reduced the growth of GBM malignant cells.

Zhou et al. (2014) used a model of subcutaneous implantation of GSC (1×10^3^ cells) in right axilla of mice [78]. After 3 weeks of GSC implantation, tumors were large, ulcerative and had complete capsule. HE stained reveled cells with dark nuclei without evidences of necrosis. Intercellular heteromorphism was obvious and vascular structure was clear. Mice treated with LY 341495 intraperitoneally (1 mg/kg per day) presented a reduced tumor volume when compared to animals treated with agonist LY 379268 (1 mg/kg per day) and vehicle. This result was sustained during 10^th^, 15^th^ and 20^th^ days after cells implantation.

Ciceroni et al. (2013) have evaluated the effect of mGluR3 inhibition plus TMZ treatment on tumor growth in nude mice implanted with human GSCs in brain parenchyma [77]. All mice were subcutaneously implanted with osmotic minipumps releasing LY 341495 antagonist (3mg/kg per day for 28 days) or saline. At the same time, mice received three injections of TMZ (70mg/kg, intraperitoneally) or vehicle (every day) during the 1^st^ week following minipump implantation. In one experiment, mice received drug treatments during 15 days after GSC implantation and were killed 30 days later (i.e. 45 days after cell implantation). In the other experiment, mice were treated with drugs during 45 days after GSC implantation and killed 30 days later (i.e. 75 days after cell implantation). Control mice (i.e., minipump releasing saline and vehicle injected intraperitoneally) showed presence of GSCs in brain with typical morphology of GBM. For mice killed 45 days after cell implantation, tumor cells were confined to medial portion of caudate nucleus close to wall of lateral ventricle. Mice killed at 75^th^ day showed infiltrating mass tumors in ipsilateral caudate nucleus. Moreover, tumor cells spread to ipsi- and contralateral portion of corpus callosum. Treatment of animals with LY 341495 or TMZ alone did not alter tumor growth in all analyzed brain areas. However, a combined treatment with TMZ plus LY341495 significantly reduced tumor growth in analyzed brain areas. The authors did not observe any signs of systemic toxicity or motor impairment in mice treated with LY 341495 (3 mg/kg per day). In addition, in this work mice survived to acute experiments using doses higher than 300 mg/kg of LY 341495.

Zhang et al. (2015) also demonstrated an anti-tumor activity of mGluR1 inhibition *in vivo* using a U-87 MG xenograft GBM model in nude mice [41]. After implantation of U-87 MG, tumor-bearing mice were treated every day with Bay 36-7620 (mGluR1 antagonist - 10 mg/kg) intraperitoneally during 24 days. Treatment reduced significantly tumor volume from 22^nd^ to 30^th^ days when compared to control group. In other experiment, nude mice were injected with si-mGluR1 transfected U-87 MG cells or si-control transfected U-87 MG cells. As expected, tumor in si-mGluR1 group had a smaller volume when compared to si-control group.

Association between antitumoral features and apparently non-toxic effects of mGluR antagonists *in vivo* indicates these drugs are potential candidates for an adjuvant treatment for GBM in humans.

## PATIENTS COHORT TO EVALUATE THE ROLE OF mGluR ON GLIOMA AGGRESSIVENESS

Only one study has evaluated the profile of mGluR mRNA expression in a cohort of patients with GBM (Figure 3C) [77]. It was analyzed a possible relationship between expression levels of mGluR3 and survival rate of patients with GBM undergoing surgery followed by radiotherapy and TMZ chemotherapy. The transcript of mGluR3 was measured by quantitative PCR in selected regions of tumor in a cohort of 87 patients. ‘Normal’ mGluR3 mRNA levels were defined as those measured in autoptic brain samples with no histological abnormalities. Levels of mGluR3 mRNA below normal range were detected in 42 GBM biopsies (48.3%), whereas 45 tumor samples (51.7%) presented higher mRNA levels. Kaplan-Meier (KM) survival analysis showed a prolonged survival rate for patients with tumoral mGluR3 RNA expression below normal range. Interestingly, five patients who survived longer than 36 months showed tumoral mGluR3 mRNA expression below normal range. On multivariate analysis, Karnofsky performance score and mGluR3 mRNA emerged as independent predictors for survival. Authors also stratified patients for mGluR3 expression and methylation of MGMT promoter. Group with low tumoral mGluR3 mRNA levels and methylated MGMT promoter showed a significantly higher survival rate as compared to low tumoral mGluR3 mRNA and unmethylated MGMT promoter group.

In summary, low levels of mGluR3 mRNA in tumor specimens may be a predictor for long survival rate in patients with GBM. In addition, methylation state of MGMT gene promoter influenced survival only in those patients whose GBM biopsies presented low expression of mGluR3 RNA. These data may encourage the use of mGluR3 antagonists as adjuvant drugs for treatment of GBM and suggest transcript levels of mGluR3 should be a potential predictor of GBM patients’ survival.

## CONCLUSIONs

A large number of preclinical studies have suggested that metabotropic glutamate receptors could be considered a prospective molecular target for treatment of several brain disorders, including depression [112], anxiety disorders [113], Alzheimer's disease [114], Parkinson's disease [115], and more recently malignant brain tumors. Several *in vitro* and *in vivo* studies have supported the putative involvement of mGluR-mediated signaling on progression, aggressiveness, and recurrence of malignant gliomas, which points to the notion that specific subtype-selective mGluR ligands may be considered as potential adjuvant chemotherapy for glioma treatment. Several academic groups [116–118], Pfizer [119], Roche [120, 121], Novartis [122] and Merck [123] have employed receptor structure-based design of mGluR selective negative allosteric modulators (NAMs) in their studies and ligand-receptor binding models were refined using mutagenesis and structure-activity data. Even though pharmaceutical and toxicological properties of all mGluR ligands are not yet entirely determined for humans (such as effective and maximum tolerable doses), several clinical studies on Phase I, II and III are being performed using mGluR modulators for treatment of distinct brain disorders and cancer. Addex Pharmaceuticals (Geneva, Switzerland) are recently performing a Phase I clinical trials on its mGluR5 ligand ADX48621 for the treatment of depression and anxiety (ClinicalTrials.gov Identifier: NCT02447640). Similarly, a compound LY 2140023, a prodrug of the group II metabotropic glutamate receptor agonist LY 404039, has been tested in a Phase III for the treatment of patients with schizophrenia (ClinicalTrials.gov Identifier: NCT01328093). Regarding cancer treatment, Barbara Ann Karmanos Cancer Institute in collaboration with National Cancer Institute (NCI) are performing a Phase I clinical trial to evaluated the efficacy of riluzole, a mGluR 1 blocker, into reduce the breast cancer growth (NCT00903214). If any of these compounds eventually obtain approval by the Food and Drug Administration (FDA), there will be a strong scientifically-based rationale for testing distinct group I and II mGluR antagonists in the treatment of malignant brain tumors through prospective, large-scale and randomized clinical trials.

## Abbreviations

>8-Bromo-cAMP: Non-metabolisable cAMP analogue
AC: Adenylyl cyclase
AMPAR: Ionotropic glutamate receptor AMPA
Bay 36-7620: mGluR1 antagonist
BCNU: Carmustine
bFGF: Basic fibroblast growth factor
BMP: Bone morphogenetic proteins
cAMP: Cyclic adenosine monophosphate
CD133: Stem cell marker
CNS: Central nervous system
Cys: Cystine
DAG: Diacylglycerol
DCG-IV: Group II mGluR agonist
DHPG: Group I mGluR agonist
EAAT2: Excitatory amino acid transporter 2
EAAT3: Excitatory amino acid transporter 3
EGF: Epidermal growth factor
EGFR: EGF receptor
EGLU: Selective group II antagonist
FACS: Fluorescence-activated cell sorting
FCS: Fetal calf serum
GBM: Glioblastoma
GFAP: Glial fibrillary acidic protein
GluR: Glutamate receptors
GSC: Glioma stem cells
HR: Hazard ratio
iGluR: Ionotropic glutamate receptors
IP_3_: Inositol (1,4,5)-trisphosphate
i.p.: intraperitoneal injection
JSH-23: NF-κB inhibitor
L-Glu: L-glutamate
LY 2389575: Group II mGluR antagonist
LY 294002: PI3K inhibitor
LY 341495: Group II mGluR antagonist
LY 379268: Group II mGluR agonist
MAPK: Mitogen-activated protein kinase
MEK: MAPK kinase
mGluR: Metabotropic glutamate receptors
MGMT: O6-methylguanine-DNA methyltransferase
MPEP: Selective group I antagonist
MRI: Magnetic resonance imaging
mTOR: Mammalian target of rapamycin
NBQX: AMPAR antagonist
NF-κB: Nuclear factor-κB
NMDAR: Ionotropic glutamate receptor NMDA
P70 S6K: p70 ribosomal protein S6 kinase
PCR: Polymerase chain reaction
PD 98059: MEK inhibitor
PI 828: PI3K inhibitor
PI3K: Phosphatidylinositol-4,5-bisphosphate 3-kinase
PiP_2_: Phosphatidylinositol-4,5-bisphosphate
PKC: Protein kinase C
PLC: Phospholipase C
siRNA: Small interfering RNA
SN 38: Irinotecan metabolite
TMZ: Temozolomide
UO126: MEK inhibitor
VEGF: Vascular endothelial growth factor
WHO: World Health Organization
Wortmannin: PI3K inhibitor
xCT: Cystine-glutamate antiporter
